# Particulate matter exposure potentiates SARS-CoV-2 delta variant infection by suppressing epithelial antiviral responses

**DOI:** 10.3389/fcimb.2025.1694050

**Published:** 2026-01-05

**Authors:** Supasek Kongsomros, Jiraporn Paha, Phayungsap Prasara-a, Sopita Visamol, Pinnakarn Techapichetvanich, Apisada Jiso, Kwanchanok Uppakara, Ardythe L. Morrow, Alexander W. Thorman, Somchai Chutipongtanate, Phisit Khemawoot, Arunee Thitithanyanont

**Affiliations:** 1 Chakri Naruebodindra Medical Institute, Faculty of Medicine Ramathibodi Hospital, Mahidol University, Samutprakarn, Thailand; 2 Division of Epidemiology, Department of Environmental and Public Health Sciences, University of Cincinnati College of Medicine, Cincinnati, OH, United States; 3 Department of Microbiology, Faculty of Science, Mahidol University, Bangkok, Thailand; 4 Department of Pharmaceutical Sciences, Faculty of Pharmacy, Chiang Mai University, Chiang Mai, Thailand; 5 Pediatric Translational Research Unit, Department of Pediatrics, Faculty of Medicine Ramathibodi Hospital, Mahidol University, Bangkok, Thailand; 6 Nanobiosciences Institute, University of Cincinnati, Cincinnati, OH, United States

**Keywords:** particulate matter, PM10, SARS-CoV-2, delta variant, air pollution, apoptosis, cytokine response

## Abstract

**Introduction:**

Airborne particulate matter (PM), particularly fine (PM_2.5_) and coarse (PM_10_) particles, is a major environmental health concern linked to increased respiratory morbidity and mortality. During the COVID-19 pandemic, epidemiological studies suggested that PM exposure may worsen SARS-CoV-2 infection outcomes; however, cellular mechanisms underlying this association remain incompletely understood. Here, we investigated how pre-exposure to PM_2.5_ and PM_10_ impacts SARS-CoV-2 infection dynamics in Calu-3 human epithelial cells.

**Methods:**

Calu-3 cells were pre-exposed to PM for 72 h prior to infection with either the wild-type Wuhan strain or the more virulent Delta variant for additional 48 h. Viral infection, receptor expression, apoptosis and cytokine responses were assessed.

**Results:**

PM_10_, but not PM_2.5_, enhanced Delta variant infection, increasing the proportion of infected cells by 13.7% and viral titers by 2.6-fold compared with controls. This enhancement was not attributable to changes in ACE2 receptor expression or viral entry efficiency but instead impaired antiviral defenses. PM10 pre-exposure suppressed apoptosis and reduced the expression of pro-inflammatory cytokines including IFN-γ, IP-10, and TNF-α during Delta infection.

**Discussion:**

These findings suggest that PM_10_ compromise epithelial antiviral response by dampening apoptotic cell clearance and inflammatory responses, thereby creating a cellular condition more permissive to viral replication. Our study provides a mechanistic basis by which particulate air pollution may amplify SARS-CoV-2 pathogenicity in a variant-specific manner. These results underscore further validation in physiologically relevant systems and highlight the potential public health implications of air pollution during viral pandemics.

## 1 Introduction

Particulate matter (PM) is a key component of air pollution and a leading environmental health risk globally (Anderson et al., 2012; Li T. et al., 2022). It arises from diverse anthropogenic and natural sources, including industrial emissions, vehicle exhaust, and biomass burning (Karagulian et al., 2015; Ryou et al., 2018). PM is typically categorized by aerodynamic diameter into ultrafine particles (UFP; ≤0.1 μm), fine particles (PM_2.5_; ≤2.5 μm), and coarse particles (PM_10_; ≤10 μm), and comprises a complex mixture of organic and inorganic compounds such as heavy metals, aliphatic hydrocarbons, polycyclic aromatic hydrocarbons (PAHs), and biological materials (Kim et al., 2015). Exposure to PM is associated with increased incidence of cardiopulmomary diseases and all-cause mortality (Anderson et al., 2012; Kim et al., 2015; Lelieveld et al., 2015; Arias-Pérez et al., 2020). Despite this, the mechanisms by which PM modulates host–virus interactions at the cellular level remain incompletely understood.

A growing body of epidemiological and experimental evidence implicates PM exposure in enhanced susceptibility to, and severity of, respiratory viral infections, including respiratory syncytial virus (Vandini et al., 2013; Nenna et al., 2017; Choi et al., 2022), influenza viruses (Landguth et al., 2020; Mishra et al., 2020), and, more recently, SARS-CoV-2 (Czwojdzińska et al., 2021; Chen et al., 2022; English et al., 2022). PM may enhance infection through multiple mechanisms, i.e. compromising epithelial barrier integrity via oxidative stress and inflammation, and impairing mucociliary clearance (Kayalar et al., 2024; Ma et al., 2024). In addition to epithelial barriers, airway innate immunity relies on resident macrophages, surfactant and antimicrobials in the airway surface, which together form the first line of defense against infection (Whitsett and Alenghat, 2015). PM exposure impairs macrophage function (Chen et al., 2020) and alters airway surface liquid composition, thereby weakening these defenses (Marín-Palma et al., 2023). At the cellular level, PM exposure has been shown to upregulate ACE2 and TMPRSS2, main receptors for SARS-CoV-2 entry (Sagawa et al., 2021; Zhu et al., 2021; Miyashita et al., 2023), and to dysregulate host antiviral defenses (Yang et al., 2020). PM_10_ inhibits cytokine responses (Mishra et al., 2020; Yang et al., 2020) and interferon signaling during H5N1 infection (Mishra et al., 2020), while PM_2.5_ suppresses production of key cytokines including IL-1β, IFN-β, and IL-6 during influenza infection, potentially through pyroptosis inhibition (Ma et al., 2017; Tao et al., 2020). However, the extent to which PM compromises epithelial immune responses in SARS-CoV-2 remains poorly defined.

Programmed cell death pathways, including apoptosis, necroptosis, and pyroptosis, are critical components of the host antiviral response, functioning to eliminate infected cells and limit viral dissemination (Barber, 2001). Among these, apoptosis is the predominant response in airway epithelial cells during SARS-CoV-2 infection, pyroptosis and necrosis appear minimally activated (Zhu et al., 2020; Liang et al., 2024). Notably, PM exposure has been reported to inhibit apoptosis or induce senescence-like states in lung epithelial cells, potentially impairing viral clearance and enabling viral persistence (Sánchez-Pérez et al., 2014; García-Cuellar et al., 2020). These effects suggest that apoptosis serves as a key regulatory node modulated by both environmental and viral factors. Similarly, cytokines such as TNF-α, IFN-γ, and IP-10 also play central roles in antiviral signaling and apoptosis regulation, and their suppression by PM may further compromise immune responses (Ma et al., 2017; Mishra et al., 2020; Tao et al., 2020).

SARS-CoV-2 variants exhibit distinct profiles in terms of infectivity, replication kinetics and immune evasion capacity (Hoteit and Yassine, 2022). The Delta variant, in particular, has been associated with higher viral loads and more severe clinical outcomes compared to the wild-type Wuhan strain (Samieefar et al., 2022; von Wintersdorff et al., 2022; Yang et al., 2023). Despite these differences, it remains unclear whether PM exposure differentially affects infection dynamics across SARS-CoV-2 variants and thus remains an important open question in the field.

In this study, we investigated how pre-exposure to PM_2.5_ and PM_10_ influences SARS-CoV-2 infection dynamics in Calu-3 human epithelial cells, using both the ancestral Wuhan strain and the more pathogenic Delta variant. We assessed viral infectivity and key host parameters, including ACE2 receptor expression, apoptosis signaling, and cytokine responses. Calu-3 cells were selected as a legitimate *in vitro* model of the human airway epithelium due to their polarized morphology and endogenous expression of ACE2 and TMPRSS2, which support productive SARS-CoV-2 infection (Kongsomros et al., 2021; Saccon et al., 2021). This simplified epithelial system is well suited for investigating cell-intrinsic antiviral responses (Saccon et al., 2021). Our findings offer mechanistic insight into how particulate pollution may impair epithelial antiviral defenses and modulate SARS-CoV-2 pathogenesis in a variant-specific manner.

## 2 Materials and methods

### 2.1 Particulate matter preparation

The standard particulate matter (PM) materials were obtained from the National Institute of Standards and Technology (NIST, Gaithersburg, USA), including fine particulate matter (PM_2.5_; SRM 2786) and coarse particulate matter (PM_10_; SRM 2787). Selected PAHs and trace elements in PM_10_ and PM_2.5_ are shown in **Supplementary Figures 1**, **2**, respectively. Scanning electron microscopy (SEM) images of PM_10_ and PM_2.5_ are shown in **Supplementary Figure 3**. The Stock solutions were prepared in dimethyl sulfoxide (DMSO; Sigma-Aldrich, St. Louis, MO, USA) at a concentration of 2 mg/mL and sonicated for 30 minutes. Aliquots were stored at −20°C until use. For all experiments, PM was diluted in culture medium to the indicated concentrations. In the No-PM control group, only DMSO was added as the vehicle, and all conditions contained an equivalent final DMSO concentration ≤ 0.05% v/v.

### 2.2 Scanning electron microscopy

The physical appearance of standard PM2.5 and PM10 (SRM 2786 and SRM 2787, respectively) were analyzed by field emission scanning electron microscopy (FE-SEM, SU5000; Hitachi, Japan). Prior to imaging, dust samples were mounted on aluminum SEM stubs using conductive carbon adhesive tape and sputter-coated with a thin layer of gold using a Q150R S Plus coater (Quorum^®^, UK) to reduce charging artifacts. Imaging was performed at a working distance of 6.5 mm and an accelerating voltage of 10 kV.

### 2.3 Cell lines

Calu-3 human adenocarcinoma epithelial cells (ATCC HTB-55) were cultured in DMEM/F12 medium (Gibco, USA) supplemented with 10% heat-inactivated fetal bovine serum (FBS; Gibco), 1% GlutaMAX (Gibco), and 1% penicillin–streptomycin (Gibco). Vero E6/TMPRSS2 cells (JCRB1819) were obtained from the Japanese Collection of Research Bioresources Cell Bank and maintained in low-glucose Dulbecco’s Modified Eagle Medium (DMEM; Gibco) supplemented with 10% FBS, 1 mg/mL G418 (Nacalai Tesque, Japan), and 100 U/mL penicillin–streptomycin. HEK293T cells expressing human ACE2 were cultured in high-glucose DMEM supplemented with 10% FBS, 2 mM L-glutamine, 100 U/mL penicillin–streptomycin, 100 µg/mL Normocin, and 0.5 µg/mL puromycin (InvivoGen). All cells were maintained at 37°C in a humidified incubator with 5% CO_2_.

### 2.4 Virus

The prototypic SARS-CoV-2 strain (SARS-CoV-2/01/human/Jan2020/Thailand) and the Delta variant (SARS-CoV-2/human/THA/NH657_P3/2021; GenBank: MZ815438) were isolated from clinical samples and confirmed by real-time PCR. Viruses were propagated in Vero E6/TMPRSS2 cells using low-glucose DMEM supplemented with 2% FBS, 1 mg/mL G418, and 100 U/mL penicillin–streptomycin. Infected cultures were incubated for 96 h at 37°C before harvesting viral supernatants. Viral titers were determined by 50% tissue culture infectious dose (TCID_50_) assays. All work with live virus was conducted in a certified biosafety level-3 laboratory at the Department of Microbiology, Faculty of Science, Mahidol University, Thailand.

### 2.5 TCID_50_ assay

Virus titers were determined by infecting Vero E6/TMPRSS2 cells in 96-well plates with 4-fold serial dilutions of virus-containing supernatants. Each dilution was tested in quadruplicate. After 96 hours, cytopathic effects were assessed, and TCID_50_ values were calculated using the Reed and Muench method.

### 2.6 PM pre-exposure and SARS-CoV-2 infection assay

Calu-3 cells were seeded at 5 × 10^4^ cells per well in 96-well plates and allowed to adhere overnight. Cells were then treated with PM_2.5_ or PM_10_ at a final concentration of 100 µg/mL for 72 h. Following pre-exposure, cells were infected with either the Wuhan strain or Delta variant of SARS-CoV-2 at 25 TCID_50_ per well. After a 1-h adsorption at 37°C, the inoculum was removed, and cells were washed with PBS and re-dosed with freshly prepared PM suspensions to ensure continuous exposure during infection. Infected cultures were incubated for 48 h before harvesting supernatants and lysates for downstream analysis.

### 2.7 Immunofluorescence staining for SARS-CoV-2 nucleoprotein

At 48 h post-infection, Calu-3 cells were fixed with 4% paraformaldehyde in PBS for 15 min and washed three times with PBS thoroughly to remove residual PMs to minimize potential light absorption artifacts. Cells were blocked with 1% BSA in PBS for 1 h at room temperature and incubated with rabbit anti-SARS-CoV-2 nucleoprotein (NP) monoclonal antibody (1:1,000; Sino Biological, Beijing, China) for 1 hour at 37°C. After washing with PBST, cells were incubated with Alexa Fluor 488-conjugated goat anti-rabbit IgG (1:1,000; A11034, Invitrogen) for 1 hour in the dark. Nuclei were counterstained with Hoechst 33342 (Invitrogen). Fluorescence signals were acquired using a Cytation 7 imaging system, and NP-positive cells were quantified using built-in software.

### 2.8 Focus forming assay

Viral titers in supernatants were further quantified using a focus forming assay. Vero E6/TMPRSS2 cells were seeded in 96-well plates and infected with 10-fold serial dilutions of vulture supernatant for 1 h. The inoculum was removed, and cells were overlaid with 1.2% Avicel in DMEM containing 2% FBS. After 24 hours, cells were fixed with 4% paraformaldehyde, permeabilized with 0.5% Triton X-100, and stained with rabbit anti-NP antibody (1:2,500; Sino Biological) followed by HRP-conjugated goat anti-rabbit IgG (1:1,000; Dako). Signal was developed using TrueBlue peroxidase substrate (SeraCare), and viral foci were counted and reported as focus forming units (FFU/mL).

### 2.9 Cytokine quantification

Cell culture supernatants were inactivated with 10% Triton X-100 for 1 hour at room temperature to ensure complete viral neutralization before transfer outside the BSL-3 facility, following our previously established protocol (Seephetdee et al., 2022). Cytokine levels were quantified using the MILLIPLEX Human Cytokine/Chemokine/Growth Factor Panel A (HCYTA-60K; MilliporeSigma) per the manufacturer’s instructions. Data were acquired using a Luminex MAGPIX system and analyzed with Belysa software. The concentrations of IFN-γ, TNF-α, and IP-10 were specifically assessed in this study.

### 2.10 MTT cell viability assay

Calu-3 cells were seeded at 5 × 10^4^ cells per well in 96-well plates and treated with serial dilutions of PM for 48 h. Cell viability was assessed using the MTT assay (Sigma-Aldrich). Briefly, cells were incubated with 0.5 mg/mL MTT for 4 h at 37°C. The medium was removed and then replaced with DMSO to dissolve formazan crystals, and absorbance was measured at 570 nm using a Cytation 7 cell imaging multi-mode reader (BioTek). Data were normalized to DMSO controls, and CC_50_ values were calculated using GraphPad Prism 7.

### 2.11 Quantification of nuclear fluorescence intensity

Cells were washed thoroughly to remove residual PMs and were then fixed with 4% paraformaldehyde and stained with Hoechst 33342 (Invitrogen) for nuclear visualization. Total nuclear fluorescence intensity was measured using a Cytation 7 multi-mode plate reader (BioTek) with excitation/emission settings of 361/497 nm. This bulk fluorescence measurement was used as a surrogate marker of relative cell number.

### 2.12 Western blot analysis

Following treatment or infection, Calu-3 cells were lysed in 1% RIPA buffer (Abcam) and denatured with 4× Laemmli buffer at 95°C for 10 minutes. Equal amounts of protein were resolved on 12% SDS-PAGE gels and transferred onto PVDF membranes (Bio-Rad). Membranes were blocked with 5% BSA in PBST and incubated overnight at 4°C with primary antibodies against cleaved caspase-3 (1:1,000; #9661, Cell Signaling Technology), poly (ADP-ribose) polymerase (PARP) (1:1,000; #9542, Cell Signaling Technology), or β-actin (1:2,000; ab8227, Abcam). After washing, membranes were incubated with HRP-conjugated goat anti-rabbit IgG (Dako) for 1 h at room temperature. Protein bands were detected using ECL substrate (Bio-Rad) and visualized using a Bio-Rad Chemidoc imaging system.

For ACE2 expression analysis, Calu-3 cells were treated with PM2.5, PM10, or vehicle control (no PM) for 72 h. Cell were then subjected to sample preparation as described above, and ACE2 protein levels were analyzed by Western blot using rabbit anti-ACE2 primary antibody (1:1,000; ab15348, Abcam). β-actin was used as a loading control. All band intensities were measured using ImageJ software (NIH) and results were normalized to β-actin and expressed relative to the no-PM control.

### 2.13 PM pre-exposure and SARS-CoV-2 pseudovirus entry assay

Lentivirus-based SARS-CoV-2 pseudovirus production was carried out as previously described by our group (Seephetdee et al., 2022). For the viral entry assay, HEK293T cells stably overexpressing human ACE2 were seeded at 1 × 10^4^ cells per well in white opaque 96-well plates (corning) and incubated overnight at 37°C with 5% CO_2_. Cells were then treated with PM_2.5_ or PM_10_ at a final concentration of 100 µg/mL for 72 h to model PM pre-exposure. Following PM treatment, cells were infected with SARS-CoV-2 pseudovirus at a concentration of 4 × 10^6^ relative light units (RLU)/mL. The pseudovirus was added directly to the wells and incubated for an additional 48 h to allow for viral entry and luciferase expression. Luciferase activity was quantified using the Bright-Glo Luciferase Assay System (Promega) according to the manufacturer’s instructions. Luminescence signals were measured using a Cytation 7 cell imaging multi-mode reader (BioTek).

### 2.14 Statistical analysis

All experiments were performed with three independent experiments (n = 3). For cytokine quantification, data represent four technical replicates derived from two independent experiments (n= 4). Results are presented as mean ± SEM. Statistical significance was determined using one-way ANOVA followed by Tukey’s multiple-comparison *post-hoc* test. All analyses and graphs were generated using GraphPad Prism 7 (GraphPad Software, CA, USA). A p-value < 0.05 was considered statistically significant.

## 3 Results

### 3.1 PM_10_ pre-exposure selectively enhances SARS-CoV-2 Delta variant infection in Calu-3 human epithelial cells

To determine whether PM influences SARS-CoV-2 infection, we first evaluated the cytotoxicity of PM_2.5_ and PM_10_ in Calu-3 human epithelial cells using the MTT assay. Cells were treated with two-fold serial concentrations of PM from 0.78 -100 μg/mL (Valderrama et al., 2022; Tseng et al., 2024) for 72 h to mimic pre-infection environmental exposure prior to viral challenge. In the absence of viral infection, neither PM_2.5_ nor PM_10_ induced cytopathic effects, and cell viability remained about 80% at PM concentrations up to 100 µg/mL (**Supplementary Figure 4**). Accordingly, the concentration of 100 µg/mL was selected for PM pre-exposure in subsequent infection experiments.

**Figure 1:**
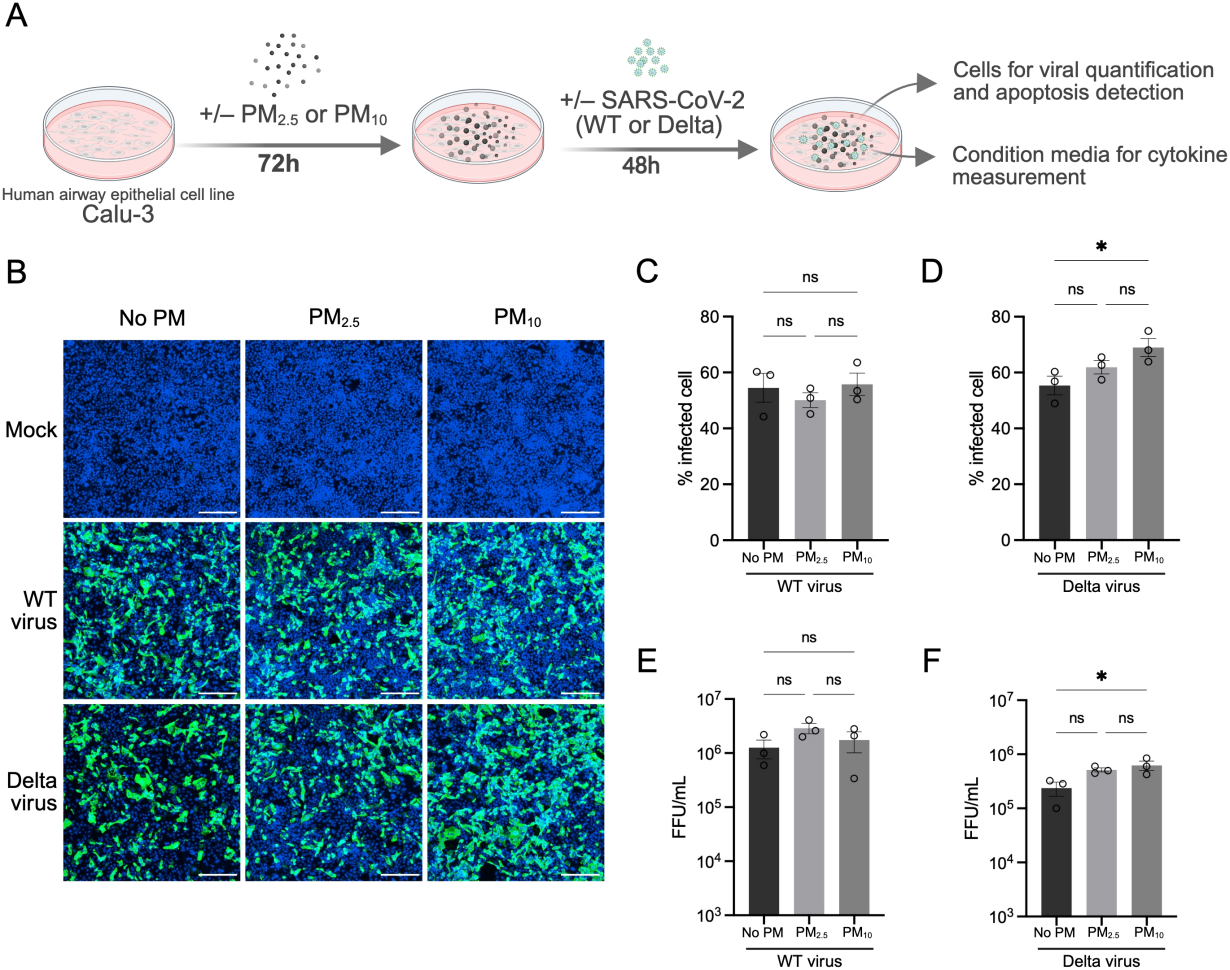
Effects of PM pre-exposure on SARS-CoV-2 infection in Calu-3 cells. **(A)** Schematic overview of the experimental design. Calu-3 cells were pre-exposed to PM_2.5_ or PM_10_ at 100 µg/mL, or non-PM exposure (No PM) for 72 h, followed by infection with either SARS-CoV-2 wildtype (WT) or Delta variant for an additional 48 (h) Cells were then fixed and immunostained for viral detection. **(B)** Representative immunofluorescence images show SARS-CoV-2-infected Calu-3 cells after PM pre-exposure. Green: viral nucleoprotein (NP) detected by anti-SARS-CoV NP monoclonal antibody; blue: nuclei stained with Hoechst. **(C, D)** Quantification of the percentage of infected cells following PM_2.5_ or PM_10_ pre-exposure for WT and Delta variants, respectively. **(E, F)** Viral titers in culture supernatants measured by foci forming assay and expressed as foci forming units (FFU)/mL for WT and Delta infections under PM_2.5_ and PM_10_ pre-exposure conditions. Data are presented as mean ± SEM from three independent biological replicates. Statistical significance was determined by one-way ANOVA followed by Tukey’s multiple comparisons test; ns, not significant; *P < 0.05.

To access the effect of PM on viral susceptibility, Calu-3 cells were pre-exposed to PM_2.5_ PM_10_, or non-PM exposure for 72 h, followed by infection with either the SARS-CoV-2 wildtype (WT virus) Wuhan strain or the more virulent Delta variant (Delta virus). After infection, cells were maintained for an additional 48 h (total experimental duration of 120 h) before fixation and immunostaining for the viral nucleoprotein antigen to quantify infection levels (**Figure 1A**). Quantitative immunofluorescence image analysis revealed that pre-exposure to PM_2.5_ did not significantly alter infection levels for either WT or Delta variants compared to untreated controls (**Figures 1B–D**). In contrast, PM_10_ pre-exposure was associated with increased Delta variant infection, as evidenced by 13.7% higher nucleoprotein-positive cells (**Figures 1B–D**). This enhancement was specific to the Delta variant and was not observed for the WT strain (**Figures 1B–D**). To further validate these findings, we measured infectious viral particles in the culture supernatants using a foci forming assay. Consistent with immunofluorescence data, PM_10_ pre-exposure resulted in a significant increase in Delta variant viral titers compared to the untreated control (mean ± SEM; 6.25 ± 1.23) × 10^5^ vs. (2.37 ± 0.69) × 10^5^ FFU/mL, p<0.05) (**Figure 1F****).** Collectively, these data suggest that pre-exposure to PM_10_, but not PM_2.5_, selectively enhances SARS-CoV-2 Delta variant infection in Calu-3 human epithelial cells.

### 3.2 Particulate matter pre-exposure does not alter ACE2 receptor expression or enhance SARS-CoV-2 viral entry

**Figure 2:**
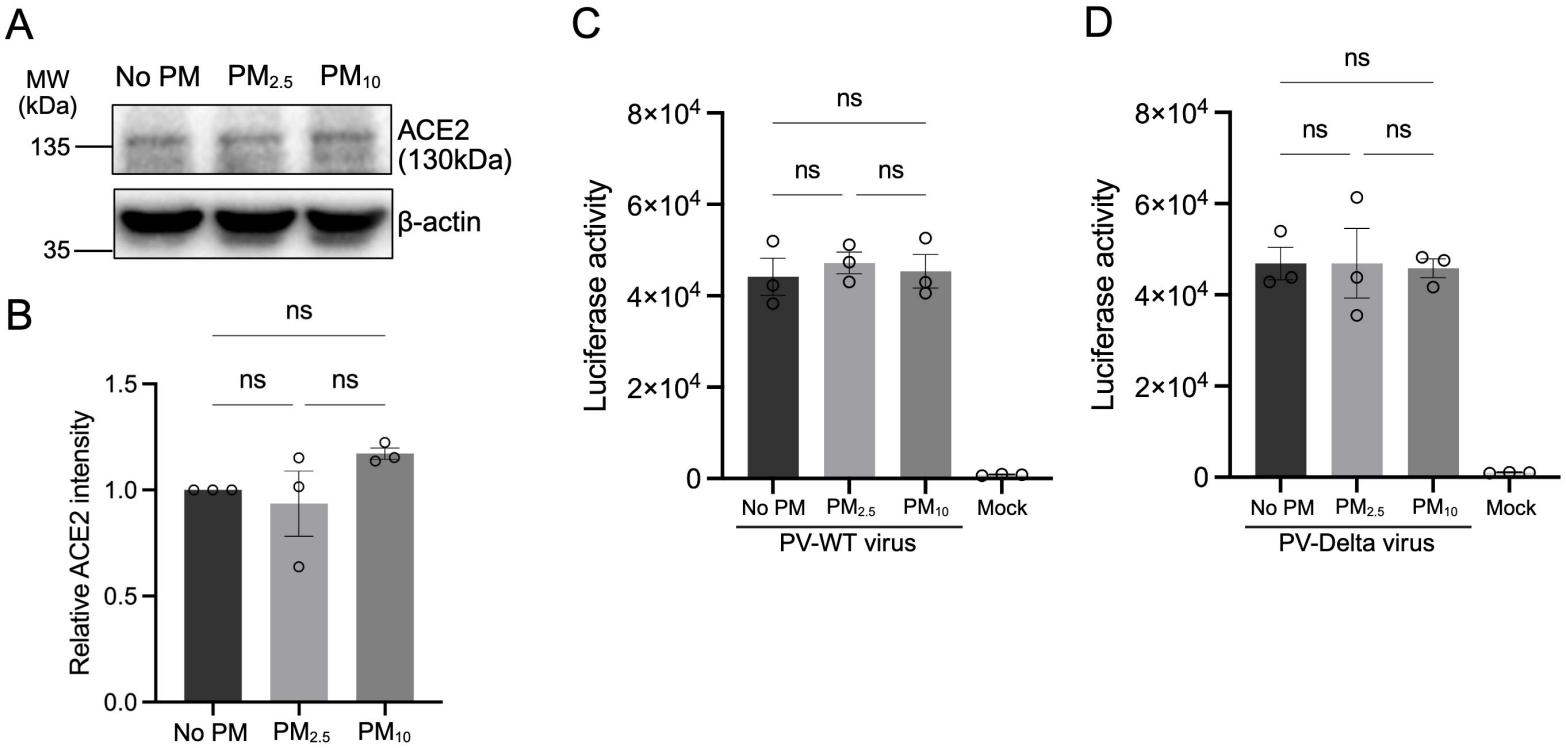
PM pre-exposure does not alter ACE2 expression or SARS-CoV-2 pseudovirus entry. **(A)** Calu-3 cells were treated with PM₂.₅, PM₁₀, or no-PM exposure control for 72 h, and ACE2 protein levels were assessed by Western blot. **(B)** Quantification of ACE2 protein expression normalized to β-actin and relative to no-PM control. **(C)** Cells were pre-exposed to PM as described in Figure 1A, then infected with lentiviral pseudoparticles bearing the SARS-CoV-2 WT spike protein and encoding a luciferase reporter. **(D)** Cells were infected with lentiviral pseudoparticles bearing the Delta spike protein. Luciferase activity was measured in cell lysates 48 h post-infection to quantify viral entry. Data are shown as mean ± SEM from three independent experiments. Statistical analysis was performed using one-way ANOVA with Tukey’s multiple comparisons test. ns, not significant.

To determine whether PM_10_-mediated enhancement of Delta variant infection is driven by changes in viral entry, we assessed expression of the entry receptor ACE2 by Western immunoblot and quantified viral entry using a spike-pseudotyped lentiviral system. Calu-3 cells were treated with PM_2.5_ or PM_10_ at 100 µg/mL for 72 h. Western blot analysis showed no significant change in ACE2 protein levels following either PM treatment (**Figures 2A, B**). We further examined the effect of PM on viral entry using a lentiviral pseudovirus system expressing the SARS-CoV-2 spike protein from either the WT or Delta variant, carrying a luciferase reporter. Calu-3 cells were pre-exposed to PM under the same conditions as live virus experiments and subsequently infected with pseudoviruses. Luciferase activity measured at 48 h post-infection showed no difference in pseudoviral entry following PM_2.5_ and PM_10_ pre-exposure for either WT or Delta spike-expressing particles (**Figures 2C, D**). These results indicate that PM_10_-mediated enhancement of Delta variant infection is not attributable to upregulated ACE2 receptor expression or facilitated viral entry, suggesting that the enhancement effect occurs at post-entry stages such as viral replication and release.

### 3.3 PM_2.5_ and PM_10_ pre-exposure attenuates SARS-CoV-2 Delta-induced apoptosis in Calu-3 human epithelial cells

**Figure 3:**
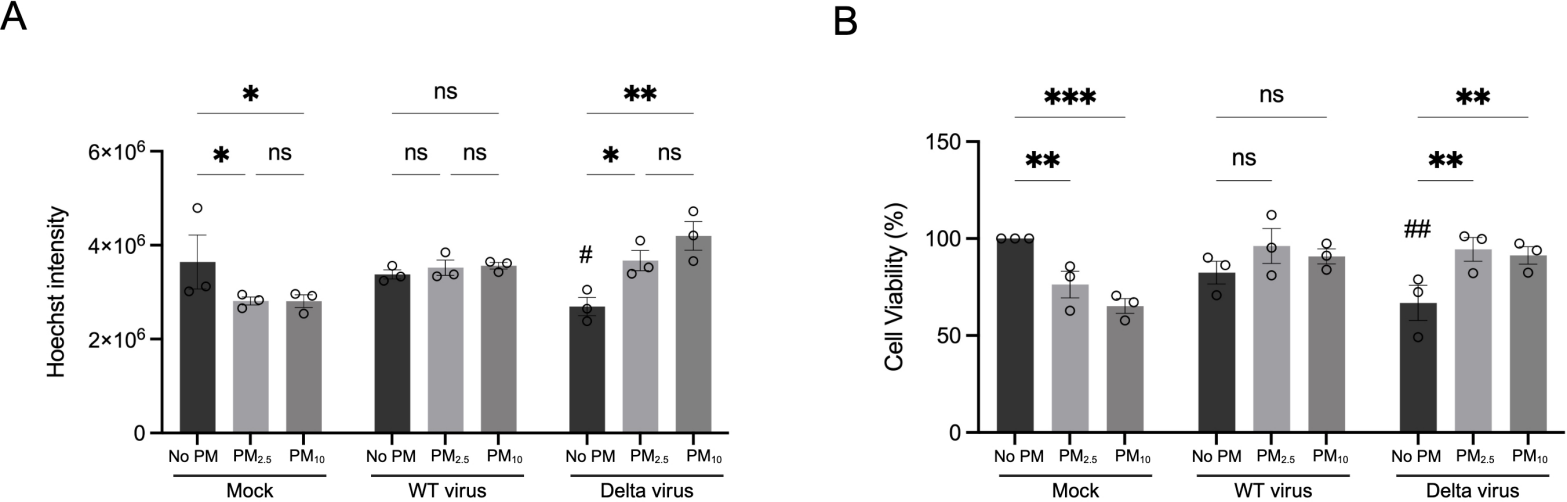
PM pre-exposure reduces SARS-CoV-2 Delta-induced total cell death in Calu-3 cells. **(A)** Quantification of nuclear Hoechst 33342 fluorescence intensity in Calu-3 cells pre-exposed to PM_2.5_, PM_10_, or no PM for 72 h, followed by infection with either SARS-CoV-2 wildtype (WT virus) or Delta variant for 48 (h) Total Hoechst fluorescence intensity was measured and used as a surrogate for relative cell number. **(B)** Cell viability was assessed by MTT assay under the same exposure and infection conditions. Cell viability was normalized to the non-infected, non-PM-exposed control group, which was set at 100%. Cell death percentage was then calculated by subtracting the viability value from 100. Data are shown as mean ± SEM from three independent experiments. Statistical significance was determined by one-way ANOVA followed by Tukey’s multiple comparisons test. ns, not significant, *P < 0.05, **P < 0.01, ***P < 0.001: compared to non-PM within the same infection group. ^#^P< 0.05, ^##^P < 0.01: compared to non-infected, non-PM control.

We next investigated whether PM pre-exposure modulates host cell responses to SARS-CoV-2 infection, focusing on virus-induced cytotoxicity. Previous studies have reported that PM_10_ can impair antiviral immunity, including suppressing pathways governing programmed cell death (Mishra et al., 2020). Interestingly, we observed an apparent reduction in Hoechst-nuclear fluorescence intensity in Delta-infected cells that were unexposed to PM compared with those pre-exposed to PM (**Figure 1B**, Delta panel), suggesting that PM may mitigate Delta-induced cytotoxicity. To test this hypothesis, we conducted the experiments described in **Figure 1A**. Following infection, nuclei were stained with Hoechst 33342, and total nuclear fluorescence intensity was quantified as a surrogate for cell number. In non-infected conditions, prolonged exposure to PM_2.5_ and PM_10_ (total of 120 h, 72 h pre-infection plus 48 h post-infection) led to a reduction in nuclear fluorescence intensity, indicating additional cell loss over time (**Figure 3A**, Mock). In WT strain, PM exposure had no significant effect on nuclear intensity compared with non-exposed groups (**Figure 3A**, WT). In contrast, infection with the Delta variant markedly reduced nuclear fluorescence (^#^P < 0.05). Cells infected with the Delta variant that were pre-exposures to PM_2.5_ or PM_10_ had significantly nuclear fluorescence intensity compared to those that were not exposed to PM (**Figure 3A**, Delta), suggesting that PM pre-exposure reduced virus-induced cytotoxicity.

To validate these findings, we assessed cell viability using MTT assay, which measures mitochondrial metabolic activity. Consistent with Hoechst quantification, prolonged exposure under the non-infected condition led to a reduction in cell viability (**Figure 3B**, Mock). These data suggest that cells may be undergoing apoptosis with disruption of mitochondrial function (Ricci et al., 2004). Infection with the WT strain moderately altered cell viability compared to non-infected controls (~20% decreased), and PM pre-exposure had minimal additional effect (**Figure 3B**, WT). Consistent with previous reports of increased cytopathic effects with Delta variant (Guo et al., 2022; Tanneti et al., 2024), infection with Delta variant significantly decreased cell viability (^##^P < 0.01) (**Figure 3B**, Delta). Notably, pre-exposure to either PM_2.5_ or PM_10_ significantly mitigated Delta-induced cell death compared to those without PM exposure (**P < 0.01, **Figure 3B**, Delta), supporting the interpretation that PM reduces Delta-induced cytotoxicity.

**Figure 4:**
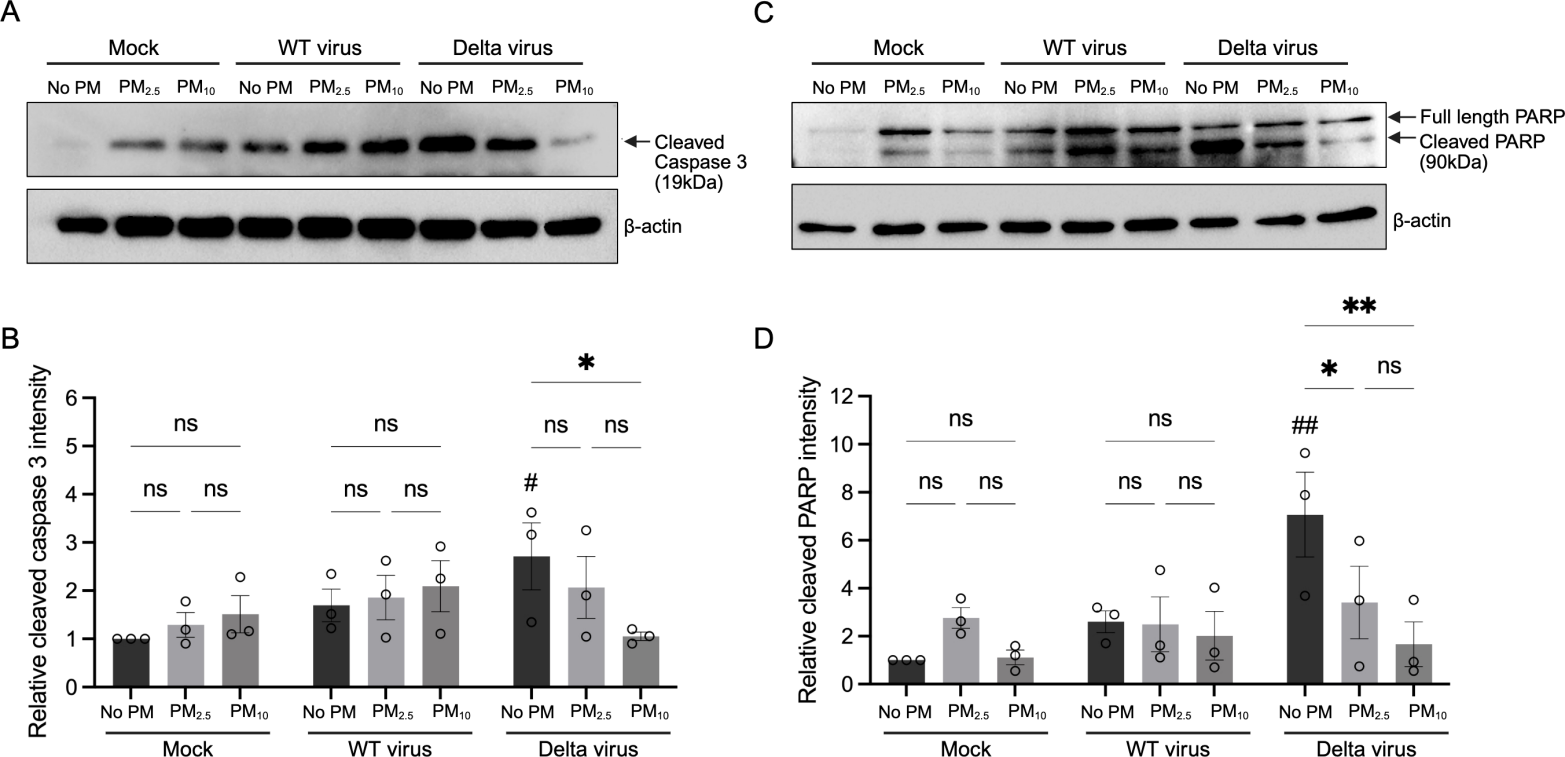
PM pre-exposure suppresses Delta-induced apoptosis in Calu-3 cells. **(A)** Representative Western blot showing cleaved caspase-3 expression in Calu-3 cells pre-exposed to PM_2.5_, PM_10_, or no PM for 72 h, followed by SARS-CoV-2 Delta variant infection for 48 (h) **(B)** Quantification of cleaved caspase-3 band intensities normalized to β-actin and expressed relative to the non-infected, non-PM control group. **(C)** Representative Western blot showing full-length and cleaved PARP expression under the same experimental conditions. **(D)** Quantification of cleaved PARP band intensities. Data are presented as mean ± SEM from three independent experiments. Statistical significance was determined by one-way ANOVA followed by Tukey’s multiple comparisons test. ns, not significant, *P < 0.05, **P < 0.01: compared to non-PM within the same infection group. ^#^P < 0.05, ^##^P < 0.01: compared to non-infected, non-PM control.

To further investigate the mechanism of SARS-CoV-2 Delta-induced cell death, we evaluated apoptosis in Calu-3 cells by assessing the expression of two key makers of apoptosis signaling, cleaved caspase-3 and cleaved poly (ADP-ribose) polymerase (PARP) (Ward et al., 2008). Western blot analysis showed that infection with Delta variant, but not the WT strain, significantly increased levels of both cleaved caspase-3 and cleaved PARP when unexposed to PM compared to non-infected, non-PM-exposed controls (**Figures 4A, D**), consistent with SARS-CoV-2-induced human lung epithelial cell apoptosis (Zhu et al., 2020; Liang et al., 2024). Interestingly, PM pre-exposure selectively attenuated this apoptotic response in Delta infection. Both cleaved caspase-3 and PARP levels were markedly reduced in PM pre-exposed cells infected with Delta infected cells compared to Delta infection alone, with the suppression being more pronounced following PM_10_ pre-exposure (**Figures 4B, 4D**). Together, these data indicate that PM pre-exposure, particularly to PM_10_, attenuates SARS-CoV-2 Delta-induced apoptosis in Calu-3 cells, potentially contributing to enhanced viral replication and viral persistence by limiting host cell clearance.

### 3.4 Pre-exposure to PM2.5 and PM10 attenuates pro-inflammatory cytokine responses during SARS-CoV-2 Delta variant infection.

**Figure 5:**
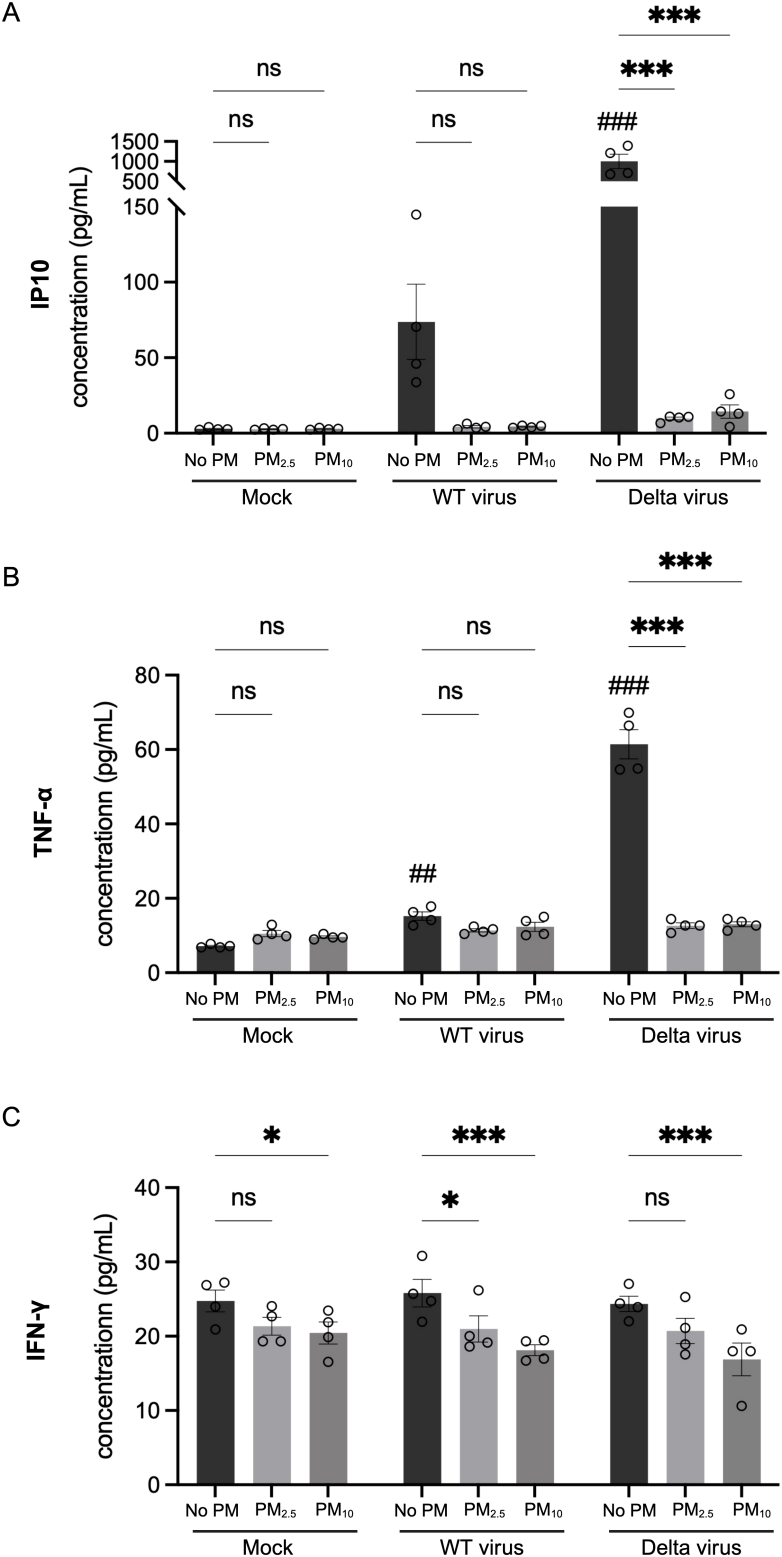
PM pre-exposure reduces pro-inflammatory cytokine responses during SARS-CoV-2 infection. **(A)** IP-10, **(B)** TNF-α, and **(C)** IFN-γ concentrations were measured by multiplex immunoassay in Calu-3 cell culture supernatants following pre-exposure to PM_2.5_, PM_10_, or non-PM exposure for 72 h, then infected with either SARS-CoV-2 wild-type (WT) or Delta variant for 48 (h) Data are presented as mean ± SD (n=4) from two independent experiments. Statistical significance was determined using one-way ANOVA followed by Tukey’s multiple comparisons test. ns, not significant. *P < 0.05, ***P < 0.001: compared to non-PM within the same infection group. ^##^P < 0.01, ^###^P < 0.001: compared to non-infected, non-PM control.

We next tested whether PM pre-exposure modulates the epithelial inflammatory response to SARS-CoV-2 infection. Cytokine levels in Calu-3 cell supernatants were measured using immunoassays following infection with either SARS-CoV-2 WT or Delta variant. Without PM pre-exposure, Delta-infected cells induced robust secretion of TNF-α and IP-10 compared to non-infected controls (^###^P < 0.01). However, cells pre-exposure to PM_2.5_ or PM_10_ showed lower levels of these cytokines relative to non-PM condition (**Figures 5A, B**). While WT infection also elevated TNF-α and IP-10 levels, PM pre-exposure did not result in a significant change (**Figures 5A, B**), suggesting strain-specific differences in PM-mediated host epithelial responses. Interestingly, IFN-γ levels remained relatively unchanged following WT or Delta infection alone, but were significantly reduced by PM_10_ pre-exposure during viral infections (**Figure 5C**). A similar downward trend was observed in the PM_2.5_ pre-exposure condition (**Figure 5C**). These results suggest that PM pre-exposure dampens key innate antiviral responses of human lung epithelial cells to SARS-CoV-2, particularly during Delta variant infection.

## 4 Discussion

Air pollution has emerged as a critical environmental factor influencing susceptibility and severity of respiratory viral infections, including SARS-CoV-2. In this study, we provide *in vitro* evidence that pre-exposure to PM_10_, but not PM_2.5_, selectively enhances infection by the SARS-CoV-2 Delta variant in human epithelial cells (**Figure 1**). This effect was not observed with the Wuhan prototypic strain and was not attributable to changes in ACE2 receptor expression or viral entry (**Figure 2**), suggesting involvement of post-entry mechanisms. To confirm receptor-mediated entry, we used a lentiviral pseudotype assay as a mechanistic control to specifically examine ACE2-dependent viral entry independent of viral replication, confirming that the observed enhancement was unlikely driven by altered receptor usage. Consistent with previous report showing that PM_10_ and PM_2.5_ do not alter ACE-2 but affect cellular responses to SARS-CoV-2 infection (Shahbaz et al., 2024). Our findings support this possibility and highlight the importance of alternative host-virus interaction pathways influenced by PM pollution.

The increase in Delta infection following PM_10_ exposure may reflect indirect effects on host cell viability or antiviral responses that render cells more permissive to viral replication. We demonstrate that PM_10_ pre-exposure suppressed epithelial antiviral responses, including apoptosis (**Figures 3**, **4**) and pro-inflammatory cytokine production (**Figure 5**), creating a cellular state that favors viral replication. These findings highlight the need to explore alternative pathways by which PM exposure compromise host resistance to viral infections.

Although PMs are considered pro-inflammatory, largely releasing from the activation of immune cells (Lim and Kim, 2024), cytokine induction may vary across experimental models. Prior studies in airway epithelial cells have reported PM-induced secretion of MIP-3α/CCL20, GM-CSF, IL-1α, and IL-8 under non-infectious conditions (Ohtoshi et al., 1998; Fujii et al., 2001; Reibman et al., 2003). In contrast, our Calu-3 model exhibited minimal cytokine induction in mock conditions, likely due to differences in particle composition, exposure context, and epithelial responsiveness. Airway epithelial cells mainly release alarmins (e.g., IL-25, IL-33, TSLP) that stimulate immune cells to produce additional inflammatory cytokines (Duchesne et al., 2022; Pham and Kim, 2025). Because our Calu-3 monocultures lack macrophages that amplify epithelial–immune crosstalk, future studies using multicellular cultures are warranted to better define how PM exposure modulates cytokine responses.

Apoptosis is a critical antiviral defense mechanism that limits viral spread by triggering programmed elimination of infected cells (Barber, 2001). SARS-CoV-2 infection has been shown to induce apoptosis in airway epithelial cells (Zhu et al., 2020; Liang et al., 2024), and in our model, Delta infection robustly induced cleaved caspase-3 and PARP (**Figure 4**). However, pre-exposure to PM_10_ significantly reduced levels of these apoptotic markers, consistent with a suppression of virus-induced apoptosis. This aligns with previous reports demonstrating that PM_10_ activates anti-apoptotic signaling pathways, such as PI3K/AKT/FoxO3a and promotes a senescence-like, apoptotic-resistant phenotype of lung epithelial cells under oxidative stress or apoptotic stimuli (Sánchez-Pérez et al., 2014; García-Cuellar et al., 2020). In parallel, PM_10_ pre-exposure was associated with lower levels of TNF-α, IFN-γ, and IP-10 during Delta variant infection (**Figure 5**). These cytokines are essential for antiviral signaling and immune recruitment (Qudus et al., 2023); TNF-α and IFN-γ synergistically promote apoptosis (Karki et al., 2021), while IP-10 limit viral replication through p53-mediated apoptotic cell death (Zhang et al., 2005). These anti-apoptotic effects may help explain the enhancement of Delta infection under PM_10_ exposure, suggesting that the attenuation of virus-induced cytotoxicity by PM_10_ may confer an evolutionary advantage to the virus, as prolonged host-cell survival could sustain replication and increase viral output. These findings are similar to previous studies in influenza virus infection models, where PM_10_ inhibited IFN responses during H5N1 infection (Mishra et al., 2020), and PM_2.5_ reduced IL-1β and IFN-β production via pyroptosis inhibition (Tao et al., 2020). Our study extends this paradigm to SARS-CoV-2, suggesting that PM air pollution broadly impair host innate antiviral defense across respiratory viruses. Interestingly, the immunosuppressive effects of PM_10_ were specific to the Delta variant. The Wuhan strain did not exhibit enhanced replication or suppressed cell death under the same conditions. This variant-specific response may reflect intrinsic differences in viral pathogenesis. The Delta variant is characterized by stronger cytopathic effect, partial resistance to type I interferon responses, and expressing viral proteins such as ORF3a, ORF7b and M protein which modulate host apoptosis and immune signaling (Guo et al., 2022; Li X. et al., 2022; Tandel et al., 2022; Tanneti et al., 2024). These properties may allow the Delta variant, but not the Wuhan strain, to exploit the altered cellular state induced by PM_10_, further enhanced viral replication and impairing host defense.

Unlike PM_10_, PM_2.5_ did not enhance Delta infection or suppress cell death and cytokine production to the same level. These differential effects may be attributable to chemical differences. Compositional analysis showed that PM_10_ (SRM 2787) contains higher levels of redox-active metals, such as aluminum and cadmium (**Supplementary Figure 1**), and polycyclic aromatic hydrocarbons (PAHs), including fluoranthene, pyrene, retene, benzo[ghi]fluoranthene, and chrysene, compared to PM_2.5_ (SRM 2786) (**Supplementary Figure 2**). Both metals and PAHs are known to modulate oxidative stress, apoptosis signaling, and immune pathways (Øvrevik et al., 2015; Pardo et al., 2020), suggesting that immunomodulatory effects of PM may be driven not only by particle size but also by specific chemical components. Heavy metals enriched in PM_10_ may influence the same ROS and apoptosis pathways that SARS-CoV-2 exploits for its replication cycle (Koyama et al., 2024; Xie et al., 2024). Thus, PM_10_ exposure could potentially interact with or partially compete with viral replication mechanisms, leading to reduced cell death and a preservation of host-cell viability. Moreover, SARS-CoV-2 has been shown to activate the Aryl hydrocarbon receptor (AhR) signaling, which facilitates viral replication (Giovannoni and Quintana, 2021; Shi et al., 2023). AhR can also be activated by PM components such as PAHs, known as AhR agonists (Torti et al., 2021). Therefore, elevated levels of PAHs may further enhance AhR activation and promote viral replication, consistent with our observations. Future studies dissecting the contributions of individual PM constituents in modulating antiviral responses and identify those components that most strongly impair host defense mechanisms.

This study is limited by the use of a single *in vitro* epithelial cell line. Although Calu-3 cells are a well-established and physiological relevant system for SARS-CoV-2 studies (Kongsomros et al., 2021; Saccon et al., 2021), this simplified model does not capture the multicellular complexity of the airway microenvironment, including interactions with immune and stromal cells. In addition, aerosol transport and deposition in the airways, along with mucus clearance and infection kinetics, also influence and contribute to SARS-CoV-2 infection (Chakravarty et al., 2025). Thus, infection kinetics, inflammatory or antiviral signaling may differ from *in vivo* conditions and the model may not fully recapitulate physiological airway responses. Therefore, our findings should be interpreted as providing mechanistic rather than physiological insights. In addition to epithelial-intrinsic effects, PM may also compromise other innate defense mechanisms in the airway including antimicrobials and alveolar macrophages. Previous studies have shown that PM exposure impairs macrophage phagocytic activity and alters airway surface liquid composition, which may further weaken mucosal defenses (Chen et al., 2020; Marín-Palma et al., 2023). These disruptions could synergize with epithelial dysfunction to enhance viral replication and persistence. Future studies using primary human airway epithelial cultures grown at the air–liquid interface (ALI), co-culture systems incorporating immune, stromal, or mucus-secreting cells to better mimic the mucosal environment, or *in vivo* animal models will be essential to establish the pathophysiological relevance of PM-mediated modulation of SARS-CoV-2 infection.

Our findings demonstrate that PM10 pre-exposure renders human lung epithelial cells more permissive to SARS-CoV-2 Delta infection by suppressing apoptosis and dampening pro-inflammatory cytokine production. These effects were not observed with PM2.5 or the Wuhan strain, suggesting a combined influence of particle composition and viral genetic background. Our study provides a mechanistic insight into how environmental pollutants can modulate host-virus interactions at the cellular level and highlight the potential impact of air quality on respiratory virus susceptibility and disease outcome.

## Data Availability

The datasets presented in this study can be found in online repositories. The names of the repository/repositories and accession number(s) can be found in the article/**Supplementary Material**.

## References

[B1] Anderson J. O. Thundiyil J. G. Stolbach A. (2012). Clearing the air: a review of the effects of particulate matter air pollution on human health. J. Med. Toxicol. 8, 166–175. doi: 10.1007/s13181-011-0203-1, PMID: 22194192 PMC3550231

[B6] Arias-Pérez R. D. Taborda N. A. Gómez D. M. Narvaez J. F. Porras J. Hernandez J. C. (2020). Inflammatory effects of particulate matter air pollution. Environ. Sci. pollut. Res. Int. 27, 42390–42404. doi: 10.1007/s11356-020-10574-w, PMID: 32870429

[B27] Barber G. N. (2001). Host defense, viruses and apoptosis. Cell Death Differ. 8, 113–126. doi: 10.1038/sj.cdd.4400823, PMID: 11313713

[B64] Chakravarty A. Kundu D. Panchagnula M. V. Mohan A. Patankar N. A. (2025). Perspectives on physics-based one-dimensional modeling of lung physiology. Front. Physiol. 16, 1635983. doi: 10.3389/fphys.2025.1635983, PMID: 41070143 PMC12504317

[B19] Chen Y. W. Huang M. Z. Chen C. L. Kuo C. Y. Yang C. Y. Chiang-Ni C. . (2020). PM(2.5) impairs macrophage functions to exacerbate pneumococcus-induced pulmonary pathogenesis. Part Fibre Toxicol. 17, 37. doi: 10.1186/s12989-020-00362-2, PMID: 32753046 PMC7409448

[B14] Chen Z. Sidell M. A. Huang B. Z. Chow T. Eckel S. P. Martinez M. P. . (2022). Ambient air pollutant exposures and COVID-19 severity and mortality in a cohort of patients with COVID-19 in southern california. Am. J. Respir. Crit. Care Med. 206, 440–448. doi: 10.1164/rccm.202108-1909OC, PMID: 35537137 PMC12039156

[B9] Choi S. Kim E. M. Kim S. Y. Choi Y. Choi S. Cho N. . (2022). Particulate matter exposure exacerbates cellular damage by increasing stress granule formation in respiratory syncytial virus-infected human lung organoids. Environ. pollut. 315, 120439. doi: 10.1016/j.envpol.2022.120439, PMID: 36257563

[B13] Czwojdzińska M. Terpińska M. Kuźniarski A. Płaczkowska S. Piwowar A. (2021). Exposure to PM2.5 and PM10 and COVID-19 infection rates and mortality: A one-year observational study in Poland. BioMed. J. 44, S25–s36. doi: 10.1016/j.bj.2021.11.006, PMID: 34801766 PMC8603332

[B50] Duchesne M. Okoye I. Lacy P. (2022). Epithelial cell alarmin cytokines: Frontline mediators of the asthma inflammatory response. Front. Immunol. 13, 975914. doi: 10.3389/fimmu.2022.975914, PMID: 36311787 PMC9616080

[B15] English P. B. Von Behren J. Balmes J. R. Boscardin J. Carpenter C. Goldberg D. E. . (2022). Association between long-term exposure to particulate air pollution with SARS-CoV-2 infections and COVID-19 deaths in California, U.S.A. Environ. Adv. 9, 100270. doi: 10.1016/j.envadv.2022.100270, PMID: 35912397 PMC9316717

[B47] Fujii T. Hayashi S. Hogg J. C. Vincent R. Van Eeden S. F. (2001). Particulate matter induces cytokine expression in human bronchial epithelial cells. Am. J. Respir. Cell Mol. Biol. 25, 265–271. doi: 10.1165/ajrcmb.25.3.4445, PMID: 11588002

[B30] García-Cuellar C. M. Chirino Y. I. Morales-Bárcenas R. Soto-Reyes E. Quintana-Belmares R. Santibáñez-Andrade M. . (2020). Airborne particulate matter (PM(10)) inhibits apoptosis through PI3K/AKT/foxO3a pathway in lung epithelial cells: the role of a second oxidant stimulus. Int. J. Mol. Sci. 21. doi: 10.3390/ijms21020473, PMID: 31940823 PMC7014458

[B62] Giovannoni F. Quintana F. J. (2021). SARS-CoV-2-induced lung pathology: AHR as a candidate therapeutic target. Cell Res. 31, 1–2. doi: 10.1038/s41422-020-00447-9, PMID: 33262451 PMC7705403

[B42] Guo K. Barrett B. S. Morrison J. H. Mickens K. L. Vladar E. K. Hasenkrug K. J. . (2022). Interferon resistance of emerging SARS-CoV-2 variants. Proc. Natl. Acad. Sci. U. S. A. 119, e2203760119. doi: 10.1073/pnas.2203760119, PMID: 35867811 PMC9371743

[B32] Hoteit R. Yassine H. M. (2022). Biological properties of SARS-coV-2 variants: epidemiological impact and clinical consequences. Vaccines (Basel) 10. doi: 10.3390/vaccines10060919, PMID: 35746526 PMC9230982

[B4] Karagulian F. Belis C. A. Dora C. F. C. Prüss-Ustün A. M. Bonjour S. Adair-Rohani H. . (2015). Contributions to cities’ ambient particulate matter (PM): A systematic review of local source contributions at global level. Atmospheric Environment 120, 475–483. doi: 10.1016/j.atmosenv.2015.08.087

[B53] Karki R. Sharma B. R. Tuladhar S. Williams E. P. Zalduondo L. Samir P. . (2021). Synergism of TNF-α and IFN-γ Triggers inflammatory cell death, tissue damage, and mortality in SARS-coV-2 infection and cytokine shock syndromes. Cell. 184, 149–68.e17. doi: 10.1016/j.cell.2020.11.025, PMID: 33278357 PMC7674074

[B17] Kayalar Ö. Rajabi H. Konyalilar N. Mortazavi D. Aksoy G. T. Wang J. . (2024). Impact of particulate air pollution on airway injury and epithelial plasticity; underlying mechanisms. Front. Immunol. 15, 1324552. doi: 10.3389/fimmu.2024.1324552, PMID: 38524119 PMC10957538

[B5] Kim K.-H. Kabir E. Kabir S. (2015). A review on the human health impact of airborne particulate matter. Environ. Int. 74, 136–143. doi: 10.1016/j.envint.2014.10.005, PMID: 25454230

[B37] Kongsomros S. Suksatu A. Kanjanasirirat P. Manopwisedjaroen S. Prasongtanakij S. Jearawuttanakul K. . (2021). Anti-SARS-coV-2 activity of extracellular vesicle inhibitors: screening, validation, and combination with remdesivir. Biomedicines 9. doi: 10.3390/biomedicines9091230, PMID: 34572416 PMC8465755

[B59] Koyama H. Kamogashira T. Yamasoba T. (2024). Heavy metal exposure: molecular pathways, clinical implications, and protective strategies. Antioxidants (Basel) 13. doi: 10.3390/antiox13010076, PMID: 38247500 PMC10812460

[B11] Landguth E. L. Holden Z. A. Graham J. Stark B. Mokhtari E. B. Kaleczyc E. . (2020). The delayed effect of wildfire season particulate matter on subsequent influenza season in a mountain west region of the USA. Environ. Int. 139, 105668. doi: 10.1016/j.envint.2020.105668, PMID: 32244099 PMC7275907

[B7] Lelieveld J. Evans J. S. Fnais M. Giannadaki D. Pozzer A. (2015). The contribution of outdoor air pollution sources to premature mortality on a global scale. Nature. 525, 367–371. doi: 10.1038/nature15371, PMID: 26381985

[B2] Li T. Yu Y. Sun Z. Duan J. (2022). A comprehensive understanding of ambient particulate matter and its components on the adverse health effects based from epidemiological and laboratory evidence. Part Fibre Toxicol. 19, 67. doi: 10.1186/s12989-022-00507-5, PMID: 36447278 PMC9707232

[B56] Li X. Zhang Z. Wang Z. Gutiérrez-Castrellón P. Shi H. (2022). Cell deaths: Involvement in the pathogenesis and intervention therapy of COVID-19. Signal Transduct Target Ther. 7, 186. doi: 10.1038/s41392-022-01043-6, PMID: 35697684 PMC9189267

[B28] Liang K. Barnett K. C. Hsu M. Chou W. C. Bais S. S. Riebe K. . (2024). Initiator cell death event induced by SARS-CoV-2 in the human airway epithelium. Sci. Immunol. 9, eadn0178. doi: 10.1126/sciimmunol.adn0178, PMID: 38996010 PMC11970318

[B46] Lim E. Y. Kim G. D. (2024). Particulate matter-induced emerging health effects associated with oxidative stress and inflammation. Antioxidants (Basel) 13. doi: 10.3390/antiox13101256, PMID: 39456509 PMC11505051

[B16] Ma J. Chiu Y. F. Kao C. C. Chuang C. N. Chen C. Y. Lai C. H. . (2024). Fine particulate matter manipulates immune response to exacerbate microbial pathogenesis in the respiratory tract. Eur. Respir. Rev. 33. doi: 10.1183/16000617.0259-2023, PMID: 39231594 PMC11372469

[B26] Ma J. H. Song S. H. Guo M. Zhou J. Liu F. Peng L. . (2017). Long-term exposure to PM2.5 lowers influenza virus resistance via down-regulating pulmonary macrophage Kdm6a and mediates histones modification in IL-6 and IFN-β promoter regions. Biochem. Biophys. Res. Commun. 493, 1122–1128. doi: 10.1016/j.bbrc.2017.09.013, PMID: 28887033

[B20] Marín-Palma D. Fernandez G. J. Ruiz-Saenz J. Taborda N. A. Rugeles M. T. Hernandez J. C. (2023). Particulate matter impairs immune system function by up-regulating inflammatory pathways and decreasing pathogen response gene expression. Sci. Rep. 13, 12773. doi: 10.1038/s41598-023-39921-w, PMID: 37550362 PMC10406897

[B12] Mishra R. Krishnamoorthy P. Gangamma S. Raut A. A. Kumar H. (2020). Particulate matter (PM(10)) enhances RNA virus infection through modulation of innate immune responses. Environ. pollut. 266, 115148. doi: 10.1016/j.envpol.2020.115148, PMID: 32771845 PMC7357538

[B21] Miyashita L. Foley G. Semple S. Gibbons J. M. Pade C. McKnight Á. . (2023). Curbside particulate matter and susceptibility to SARS-CoV-2 infection. J. Allergy Clin. Immunol. Glob. 2, 100141. doi: 10.1016/j.jacig.2023.100141, PMID: 37781647 PMC10509961

[B10] Nenna R. Evangelisti M. Frassanito A. Scagnolari C. Pierangeli A. Antonelli G. . (2017). Respiratory syncytial virus bronchiolitis, weather conditions and air pollution in an Italian urban area: An observational study. Environ. Res. 158, 188–193. doi: 10.1016/j.envres.2017.06.014, PMID: 28647513 PMC7125886

[B48] Ohtoshi T. Takizawa H. Okazaki H. Kawasaki S. Takeuchi N. Ohta K. . (1998). Diesel exhaust particles stimulate human airway epithelial cells to produce cytokines relevant to airway inflammation *in vitro*. J. Allergy Clin. Immunol. 101, 778–785. doi: 10.1016/S0091-6749(98)70307-0, PMID: 9648705

[B58] Øvrevik J. Refsnes M. Låg M. Holme J. A. Schwarze P. E. (2015). Activation of proinflammatory responses in cells of the airway mucosa by particulate matter: oxidant- and non-oxidant-mediated triggering mechanisms. Biomolecules. 5, 1399–1440. doi: 10.3390/biom5031399, PMID: 26147224 PMC4598757

[B57] Pardo M. Qiu X. Zimmermann R. Rudich Y. (2020). Particulate matter toxicity is nrf2 and mitochondria dependent: the roles of metals and polycyclic aromatic hydrocarbons. Chem. Res. Toxicol. 33, 1110–1120. doi: 10.1021/acs.chemrestox.0c00007, PMID: 32302097 PMC7304922

[B51] Pham D. D. Kim T. B. (2025). Epithelial-derived cytokines in the pathogenesis of severe asthma. Front. Allergy 6, 1681147. doi: 10.3389/falgy.2025.1681147, PMID: 41169790 PMC12568661

[B52] Qudus M. S. Tian M. Sirajuddin S. Liu S. Afaq U. Wali M. . (2023). The roles of critical pro-inflammatory cytokines in the drive of cytokine storm during SARS-CoV-2 infection. J. Med. Virol. 95, e28751. doi: 10.1002/jmv.28751, PMID: 37185833

[B49] Reibman J. Hsu Y. Chen L. C. Bleck B. Gordon T. (2003). Airway epithelial cells release MIP-3alpha/CCL20 in response to cytokines and ambient particulate matter. Am. J. Respir. Cell Mol. Biol. 28, 648–654. doi: 10.1165/rcmb.2002-0095OC, PMID: 12760962

[B41] Ricci J. E. Muñoz-Pinedo C. Fitzgerald P. Bailly-Maitre B. Perkins G. A. Yadava N. . (2004). Disruption of mitochondrial function during apoptosis is mediated by caspase cleavage of the p75 subunit of complex I of the electron transport chain. Cell. 117, 773–786. doi: 10.1016/j.cell.2004.05.008, PMID: 15186778

[B3] Ryou H. G. Heo J. Kim S. Y. (2018). Source apportionment of PM(10) and PM(2.5) air pollution, and possible impacts of study characteristics in South Korea. Environ. pollut. 240, 963–972. doi: 10.1016/j.envpol.2018.03.066, PMID: 29910064

[B36] Saccon E. Chen X. Mikaeloff F. Rodriguez J. E. Szekely L. Vinhas B. S. . (2021). Cell-type-resolved quantitative proteomics map of interferon response against SARS-CoV-2. iScience. 24, 102420. doi: 10.1016/j.isci.2021.102420, PMID: 33898942 PMC8056843

[B23] Sagawa T. Tsujikawa T. Honda A. Miyasaka N. Tanaka M. Kida T. . (2021). Exposure to particulate matter upregulates ACE2 and TMPRSS2 expression in the murine lung. Environ. Res. 195, 110722. doi: 10.1016/j.envres.2021.110722, PMID: 33422505 PMC7789825

[B33] Samieefar N. Rashedi R. Akhlaghdoust M. Mashhadi M. Darzi P. Rezaei N. (2022). Delta variant: the new challenge of COVID-19 pandemic, an overview of epidemiological, clinical, and immune characteristics. Acta Biomed. 93, e2022179. doi: 10.23750/abm.v93i1.12210, PMID: 35315394 PMC8972886

[B31] Sánchez-Pérez Y. Chirino Y. I. Osornio-Vargas ÁR. Herrera L. A. Morales-Bárcenas R. López-Saavedra A. . (2014). Cytoplasmic p21(CIP1/WAF1), ERK1/2 activation, and cytoskeletal remodeling are associated with the senescence-like phenotype after airborne particulate matter (PM(10)) exposure in lung cells. Toxicol. Lett. 225, 12–19. doi: 10.1016/j.toxlet.2013.11.018, PMID: 24291038

[B38] Seephetdee C. Bhukhai K. Buasri N. Leelukkanaveera P. Lerdwattanasombat P. Manopwisedjaroen S. . (2022). A circular mRNA vaccine prototype producing VFLIP-X spike confers a broad neutralization of SARS-CoV-2 variants by mouse sera. Antiviral Res. 204, 105370. doi: 10.1016/j.antiviral.2022.105370, PMID: 35772601 PMC9235288

[B45] Shahbaz M. A. Kuivanen S. Mussalo L. Afonin A. M. Kumari K. Behzadpour D. . (2024). Exposure to urban particulate matter alters responses of olfactory mucosal cells to SARS-CoV-2 infection. Environ. Res. 249, 118451. doi: 10.1016/j.envres.2024.118451, PMID: 38341073

[B61] Shi J. Du T. Wang J. Tang C. Lei M. Yu W. . (2023). Aryl hydrocarbon receptor is a proviral host factor and a candidate pan-SARS-CoV-2 therapeutic target. Sci. Adv. 9, eadf0211. doi: 10.1126/sciadv.adf0211, PMID: 37256962 PMC10413656

[B55] Tandel D. Sah V. Singh N. K. Potharaju P. S. Gupta D. Shrivastava S. . (2022). SARS-coV-2 variant delta potently suppresses innate immune response and evades interferon-activated antiviral responses in human colon epithelial cells. Microbiol. Spectr 10, e0160422. doi: 10.1128/spectrum.01604-22, PMID: 36073824 PMC9602719

[B43] Tanneti N. S. Patel A. K. Tan L. H. Marques A. D. Perera R. Sherrill-Mix S. . (2024). Comparison of SARS-CoV-2 variants of concern in primary human nasal cultures demonstrates Delta as most cytopathic and Omicron as fastest replicating. mBio. 15, e0312923. doi: 10.1128/mbio.03129-23, PMID: 38477472 PMC11005367

[B25] Tao R. J. Cao W. J. Li M. H. Yang L. Dai R. X. Luo X. L. . (2020). PM2.5 compromises antiviral immunity in influenza infection by inhibiting activation of NLRP3 inflammasome and expression of interferon-β. Mol. Immunol. 125, 178–186. doi: 10.1016/j.molimm.2020.07.001, PMID: 32717666

[B63] Torti M. F. Giovannoni F. Quintana F. J. García C. C. (2021). The aryl hydrocarbon receptor as a modulator of anti-viral immunity. Front. Immunol. 12, 624293. doi: 10.3389/fimmu.2021.624293, PMID: 33746961 PMC7973006

[B40] Tseng W.-J. Eboña K. R. Lien S.-H. Tayo L. L. Tsai J.-H. Lu J.-H. . (2024). The toxicity of human lung epithelial cells exposure to PM2.5 and glucose before or after intervention of guilu erxian jiao. Aerosol Air Qual. Res. 24, 240165. doi: 10.4209/aaqr.240165

[B39] Valderrama A. Ortiz-Hernández P. Agraz-Cibrián J. M. Tabares-Guevara J. H. Gómez D. M. Zambrano-Zaragoza J. F. . (2022). Particulate matter (PM(10)) induces *in vitro* activation of human neutrophils, and lung histopathological alterations in a mouse model. Sci. Rep. 12, 7581. doi: 10.1038/s41598-022-11553-6, PMID: 35534522 PMC9083477

[B8] Vandini S. Corvaglia L. Alessandroni R. Aquilano G. Marsico C. Spinelli M. . (2013). Respiratory syncytial virus infection in infants and correlation with meteorological factors and air pollutants. Ital J. Pediatr. 39, 1. doi: 10.1186/1824-7288-39-1, PMID: 23311474 PMC3553040

[B34] von Wintersdorff C. J. H. Dingemans J. van Alphen L. B. Wolffs P. F. G. van der Veer B. Hoebe C. . (2022). Infections with the SARS-CoV-2 Delta variant exhibit fourfold increased viral loads in the upper airways compared to Alpha or non-variants of concern. Sci. Rep. 12, 13922. doi: 10.1038/s41598-022-18279-5, PMID: 35978025 PMC9382600

[B44] Ward T. H. Cummings J. Dean E. Greystoke A. Hou J. M. Backen A. . (2008). Biomarkers of apoptosis. Br. J. Cancer 99, 841–846. doi: 10.1038/sj.bjc.6604519, PMID: 19238626 PMC2538762

[B18] Whitsett J. A. Alenghat T. (2015). Respiratory epithelial cells orchestrate pulmonary innate immunity. Nat. Immunol. 16, 27–35. doi: 10.1038/ni.3045, PMID: 25521682 PMC4318521

[B60] Xie J. Yuan C. Yang S. Ma Z. Li W. Mao L. . (2024). The role of reactive oxygen species in severe acute respiratory syndrome coronavirus 2 (SARS-COV-2) infection-induced cell death. Cell Mol. Biol. Lett. 29, 138. doi: 10.1186/s11658-024-00659-6, PMID: 39516736 PMC11549821

[B24] Yang L. Li C. Tang X. (2020). The impact of PM(2.5) on the host defense of respiratory system. Front. Cell Dev. Biol. 8, 91. doi: 10.3389/fcell.2020.00091, PMID: 32195248 PMC7064735

[B35] Yang J. H. Yang M. S. Kim D. M. Kim B. Tark D. Kang S. M. . (2023). Delta (B1.617.2) variant of SARS-CoV-2 induces severe neurotropic patterns in K18-hACE2 mice. Sci. Rep. 13, 3303. doi: 10.1038/s41598-023-29909-x, PMID: 36849513 PMC9970970

[B54] Zhang H. M. Yuan J. Cheung P. Chau D. Wong B. W. McManus B. M. . (2005). Gamma interferon-inducible protein 10 induces HeLa cell apoptosis through a p53-dependent pathway initiated by suppression of human papillomavirus type 18 E6 and E7 expression. Mol. Cell Biol. 25, 6247–6258. doi: 10.1128/MCB.25.14.6247-6258.2005, PMID: 15988033 PMC1168823

[B22] Zhu T. Y. Qiu H. Cao Q. Q. Duan Z. L. Liu F. L. Song T. Z. . (2021). Particulate matter exposure exacerbates susceptibility to SARS-CoV-2 infection in humanized ACE2 mice. Zool Res. 42, 335–338. doi: 10.24272/j.issn.2095-8137.2021.088, PMID: 33998180 PMC8175951

[B29] Zhu N. Wang W. Liu Z. Liang C. Wang W. Ye F. . (2020). Morphogenesis and cytopathic effect of SARS-CoV-2 infection in human airway epithelial cells. Nat. Commun. 11, 3910. doi: 10.1038/s41467-020-17796-z, PMID: 32764693 PMC7413383

