# Supplementary material for: Particulate matter exposure potentiates SARS-CoV-2 delta variant infection by suppressing epithelial antiviral responses
Source: Front Cell Infect Microbiol. 2026 Jan 5;15:1694050. doi: 10.3389/fcimb.2025.1694050 (PMC12813143; doi:10.3389/fcimb.2025.1694050)
Supplement: Supplementary file 1 [file Table1.docx]

Supplementary Material

# Supplementary Figures


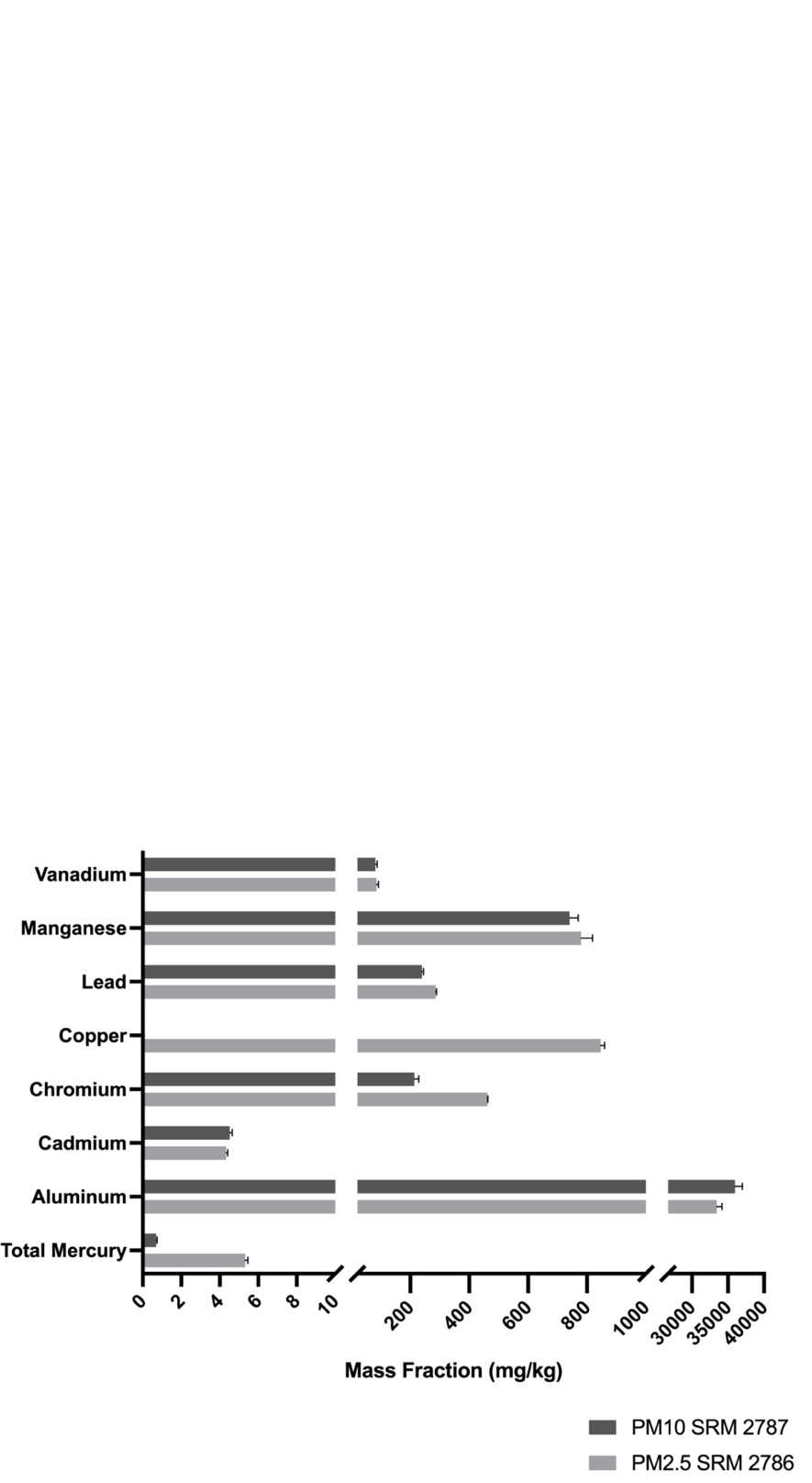


**Supplementary Figure 1**. Selected trace element composition in PM2.5 and PM10. Concentrations (mg/kg) of representative trace elements in NIST SRM 2786 (PM2.5) and SRM 2787 (PM10) are plotted. Data were extracted from the certified reference materials provided by the National Institute of Standards and Technology (NIST), USA and visualized for comparative purposes.


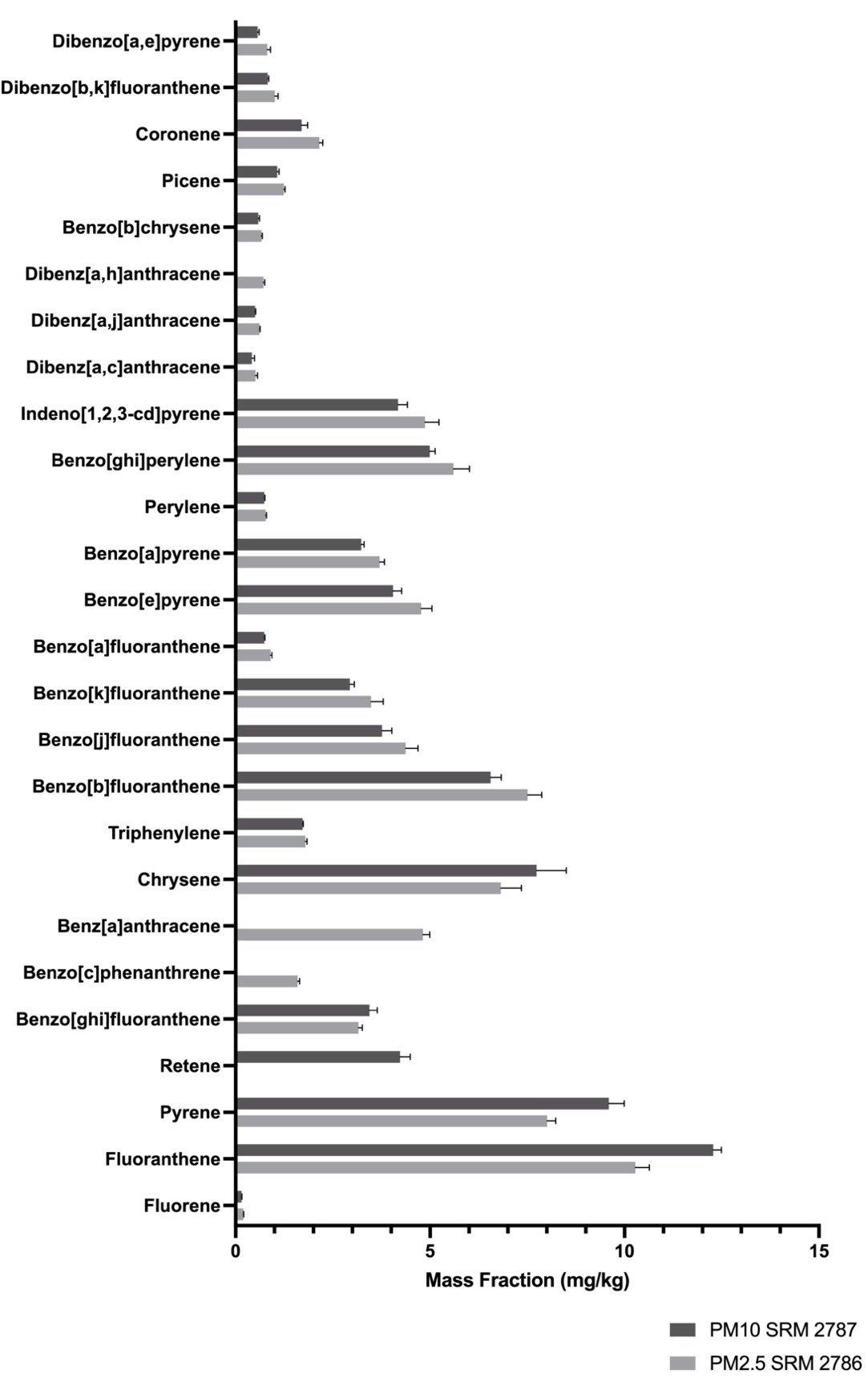


**Supplementary Figure 2**. Selected polycyclic aromatic hydrocarbon (PAH) content in PM2.5 and PM10. Concentrations (mg/kg) of representative PAHs in NIST SRM 2786 (PM2.5) and SRM 2787 (PM10) are plotted. Data were extracted from the certified reference materials provided by the National Institute of Standards and Technology (NIST), USA and visualized for comparative purposes.


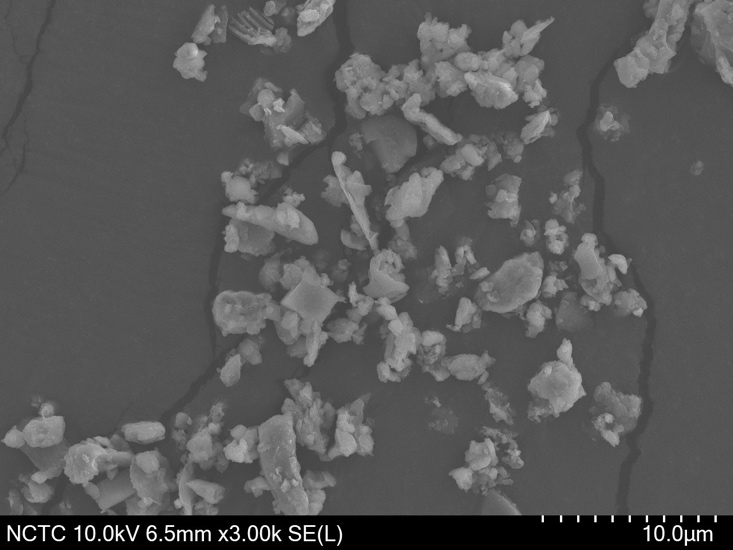
**A**


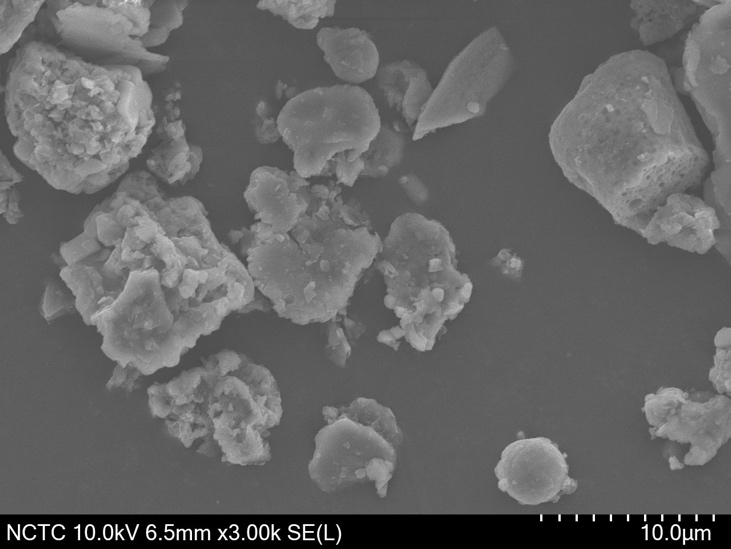
**B**

**Supplementary Figure 3.** Scanning electron microscopy (SEM) images of PM2.5 (A) and PM10 (B) showing particle morphology.

**Supplementary Figure 4. Cytotoxicity of PM2.5 and PM10 on Calu-3 cells prior infection experiments.** Calu-3 cells were seeded in a 96-well plate overnight and then treated with particulate matter (PM) 2.5 and PM10 at concentrations ranging from 0.78 to 100 µg/mL in medium for 72 h. Cell viability was assessed using the MTT assay. Absorbance was measured at a wavelength of 570 nm. Data were normalized to the DMSO control and presented as a percentage of cell viability. The dotted line at 80% represents the cytotoxicity threshold used to define non-lethal conditions. Concentrations above this cutoff were considered non-cytotoxic conditions and used for subsequent viral infection experiments.
